# Breaking and Connecting: Highly Hazy and Transparent Regenerated Networked-Nanofibrous Cellulose Films via Combination of Hydrolysis and Crosslinking

**DOI:** 10.3390/nano12152729

**Published:** 2022-08-08

**Authors:** Jamaliah Aburabie, Raed Hashaikeh

**Affiliations:** NYUAD Water Research Center, Department of Engineering, New York University Abu Dhabi, Abu Dhabi P.O. Box 129188, United Arab Emirates

**Keywords:** transparent, hazy, cellulose, crosslinking, hydrolysis, solar cells, optoelectronics

## Abstract

High optical transparency combined with high optical haze are essential requirements for optoelectronic substrates. Light scattering caused by haze is responsible for increasing light harvesting in photon-absorbing active materials, hence increasing efficiencies. A trade-off between transparency and haze is common in solar substrates with high transparency (~90%) and low optical haze (~20%), or vice versa. In this study, we report a novel, highly transparent film fabricated from regenerated cellulose after controlled acid-hydrolysis of microcrystalline cellulose (MCC). The developed networked-nanofibrous cellulose was chemically crosslinked with glutaraldehyde (GA) and vacuum-cured to facilitate the fabrication of mechanically stable films. The effects of crosslinker concentration, crosslinking time, and curing temperature were investigated. Optimum conditions for fabrication unveils high optical transparency (~94%) and high haze (~60%), using 25% GA for 24 hr with a curing temperature of 25 °C; therefore, conveying an optimal substrate for optoelectronics applications. The high haze arises primarily from the crystalline, networked crystals of cellulose II structure formed within the regenerated cellulose upon hydrolysis. Moreover, the developed crosslinked film presents high thermal stability, water resistance, and good mechanical resilience. This high-performance crosslinked cellulose film can be considered a potential material for new environmentally-friendly optical substrates.

## 1. Introduction

Global energy depletion is one of the major challenges faced in the 21st century. Exhaustible fossil fuels dominate our daily power generation and, disturbingly, 38% of the world’s electricity consumption depends on coal, according to the International Energy Agency report in 2019. The world consumption of fossil fuels witnessed increasing trends over the last half century, and is expected to keep escalating [1]. With this growth of consumption, it is predicted that reserves will soon be diminished [2]. Additionally, the environmental damage caused by burning fossil fuels, such as pollution and global warming, presents a pressing hurdle. Consequently, much of the efforts are committed to exploring alternative sources for energy. Renewable sources of energy hold boundless potential to replace fossil fuels and reduce environmental impact; solar and wind energy, to name a few. One promising viable source of green energy is solar energy; therefore, optoelectronic devices such as solar cells is being actively researched. Solar cells are photovoltaic devices that harness sunlight and convert its energy directly into electricity. Sunlight energy is free; however, the materials and available space required to use solar energy have high costs. Solar panels with low efficiency, for example, require more panels occupying more space, in order to cover the same electricity demand [3]. The thriving sales of silicon wafer-based photovoltaics almost completely dominate the market; however, silicon wafers are costly [4]. One way to eliminate major costs is by fabricating cells in the form of thin films, deposited onto substrates of different variety, such as glass and polymers [5,6,7,8]. The choice of substrate is significant for optoelectronic systems as they provide mechanical support, environmental protection, optical management, thermal stability, and moisture resistivity [9].

The light management of the substrate plays a key role in the performance of solar cells and greatly influence their efficiency. Having both high transparency and high optical haze are desirable features for solar cell applications. The percentage of optical haze is described as the percentage of transmitted light through a substrate that diffusely scatters. This advantageous scattering of sunlight increases the absorption path of light, enhancing solar cell conversion efficiency [10,11]. The market for flexible thin-film solar cells has been influenced by plastic substrates, due to their low costs and light weight. However, the mass production of electronic devices such as solar cells generates huge amounts of nonbiodegradable toxic electronic waste. To eliminate this drawback, biodegradable polymers, such as silk [12] and cellulose, have recently emerged as a substitute for plastic substrates. Unfortunately, marrying high transparency with high haze in common transparent cellulose substrates remains a challenge.

Cellulose transparent films with high haze have recently been introduced as novel sustainable substrates for solar cells. Oxidation of cellulose fibers with 2,2,6,6-tetramethylpiperidine-1-oxyl (TEMPO) is a common method to fabricate cellulose transparent and hazy substrates [13,14,15,16]. Fang et al. reported the preparation of transparent wood fiber paper, the substrates combining a high optical transparency of ~96% and a high optical haze of ~60%. Wood fibers were treated by an oxidation system of TEMPO/NaBr/NaCIO. The oxidation weakens the hydrogen bonds, causing fibers to densely pack, introducing carboxyl groups and boosting optical properties [17]. Substrates with nearly theoretical haze (95.2%) and high transparency (90.1%) were reported by Hou et al., the substrates were cellulose composite films fabricated with softwood fibers and regenerated cellulose [18]. Thin-film solar cells, such as a perovskite solar cell [19], are sensitive to humidity and suffer from degradation due to the permeation of water vapors through the substrate [20]. Therefore, substrates with high water- and humidity-resistance are needed. Hu et al. reported protonation of a composite film of paper and carboxymethyl cellulose via an acetic acid immersion and desalination process in an alcohol bath; films had high combined transparency and haze properties of 90.9 and 83.6%, respectively, with a water contact angle of 72°. Physical crosslinking among the protonated carboxymethyl cellulose chains and wood fibers enhanced water resistivity [21]. Transparent and hazy nanopaper with a superhydrophobic surface was fabricated with TEMPO-oxidized cellulose nanofibrils (TOCNF) and polysiloxane; the prepared films showed a transparency of 90.2% and haze of 46.5%, with an impressive hydrophobicity surface of a 159.6° contact angle [22]. The chemical structure of cellulose can be altered to many forms with different properties from the mother form. Modification of cellulose implements changes in morphology and crystallinity. These modifications, once regeneration takes place, are reflected in the properties of the fabricated films.

An amorphous, modified network structure (NC) with cellulose II crystals was developed via controlled acid-hydrolysis [23]. This modified, regenerated cellulose possesses an open-networked, fibrous structure formed during regeneration and recrystallization. During the regeneration stage, the NC chains arrange themselves into a network with cellulose II crystals. The open network of NC gives the cellulose high mechanical and thermal stability [24]. We believe that the formation of the networked fibrous structure with cellulose II crystals equips the NC material with light-diffusive properties. Further chemical crosslinking of such material will improve such properties as well as film fabrication. Glutaraldehyde (GA) is one of the crosslinking agents that have been heavily researched for the crosslinking of cellulose; the reason is that dialdehydes improve resilience, mechanical properties, and thermal stability [25]. For the first time, we report the fabrication of a highly transparent and hazy substrate, composed of the abundant and biodegradable cellulose via a combination of controlled acid-hydrolysis (breaking chains) and chemical crosslinking (connecting chains). The hydrophobic, transparent, and hazy crosslinked cellulose film has potential to be applied to solar cell applications in sustainable and environmentally-friendly optoelectronics devices.

## 2. Materials and Methods

### 2.1. Materials

Microcrystalline cellulose (MCC) powder was supplied by FMC BioPolymer (Avicel-PH101). Sulfuric acid (H_2_SO_4_) Reagent Grade (95–97%) was procured from VWR Chemicals-USA. Ethanol and the 25 wt.% glutaraldehyde aqueous solution were purchased from Sigma Aldrich-USA. All chemicals were used as received without further modifications.

### 2.2. Methods

#### 2.2.1. Preparation of Networked–Nanofibrous Cellulose Suspension (NC)

**Figure 1:**
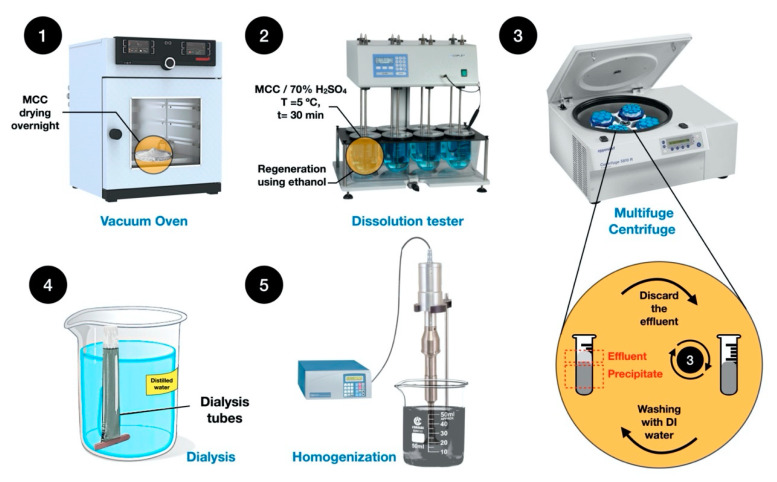
Schematic illustration of the preparation steps (from 1 to 5) for networked–nanofibrous cellulose (NC).

Networked–nanofibrous cellulose was restructured via the regeneration of acid-hydrolyzed cellulose solution. Acid hydrolysis was carried out using Copley Dissolution Tester (DIS 6000)-UK, following a procedure reported in a previous study [23]. The temperature of the water bath was fixed at 5 °C using ice packs. A 70% (*w*/*w*) diluted sulfuric acid was prepared and subsequently added, kept under cold conditions (5 °C). An amount of 10 g of MCC was mixed with 100 mL of sulfuric acid, and the solution was stirred at 200 rpm for 1 hr at 5 °C until a viscous transparent solution was formed. Ethanol (stored at 5 °C) was added to the cellulose solution as a regeneration solvent. After the addition of ethanol, the mixture was mixed for another 20 min to allow for the complete regeneration of cellulose. Following the complete generation of cellulose, centrifugation was carried out at 2400× *g* rpm at 5 °C, resulting in a separation between the cellulose-rich layer at the bottom and the liquid acidic layer on top, which was decanted. The centrifugation process was repeated three times. The bottom layer was collected and dialyzed in a Spectra/Por dialysis membrane (MWCO: 12–14 K g/mol) under running tap water (3 days) until the suspension pH reached 7–8 (tap water pH). The resulting NC and water suspension was homogenized by a mechanical homogenizer (IKA-T25 ULTRA-TURRAX). The suspension was kept in cold storage (5 °C) until further use; Figure 1 illustrates the preparation procedure. The yield of the network cellulose was calculated by freeze-drying three samples in a freeze dryer for 24 hr. The resulting suspension had a 96% yield and a 3% (*w*/*w*) concentration.

#### 2.2.2. Preparation of Crosslinked Regenerated Networked–Nanofibrous Cellulose Substrates (CRC)

**Scheme 1:**
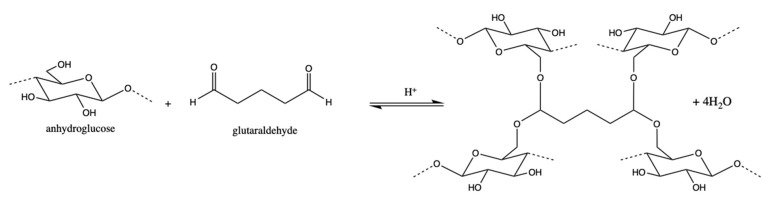
Crosslinking reaction of regenerated networked-–nanofibrous cellulose and glutaraldehyde.

A glutaraldehyde (GA) crosslinker was used to chemically crosslink the regenerated cellulose. The reaction between the dialdehyde of GA and the hydroxyl groups of cellulose is expected to form an acetal under catalyst conditions (i.e., acidic). Crosslinking occurred where every GA molecule connected with four anhydroglucose units; Scheme 1 shows the crosslinking reaction. The end mixture solutions of NC—GA were mixed at room temperature, maintaining a solution of 60% NC/water suspension and 40% GA/water. An amount of 350 μL of 20% H_2_SO_4_ was added to catalyze the reaction (pH of ~1.8). The concentrations of NC suspension was kept unchanged at 3% (*w*/*w*), whereas the concentrations of GA were varied between 6, 12, and 25%. The crosslinking reaction time was studied for 3, 6, and 24 hr. After mixing, 4 mL of the solution was poured into a petri dish and oven-cured in a vacuum oven at 25 °C, unless mentioned otherwise, for 3 hr. After drying, the crosslinked regenerated cellulose (CRC) thin film was wetted with DI water to encourage detachment from the glass; the prepared films were rinsed with water to remove excess GA and kept wet. Films were subjected to air drying when needed in a dry state.

### 2.3. Measurements and Characterizations

A Fourier-transform infrared spectrometer (FTIR) (Thermo scientific™-USA, Nicolet iS5 with iD7 ATR accessory) with the scanning wavelength range of 4000–500 cm^−1^ was used to characterize the NC and CRC films. Scanning electron microscope (SEM) images were captured using the ThermoFischer™ Quanta 450 FEG, USA. Microstructure images were obtained using ThermoFischer™ Talos F200X, USA Transmission Electron Microscopy (TEM). Samples were prepared by applying a drop of the suspension on Agar Scientific^®^ holey carbon S147-4 grids and allowing to air dry. Contact angle measurements on the membrane surface was performed using an EasyDrop Standard drop shape analysis (DSA100, Krüss GmbH, Germany). A deionized water droplet with a volume of 2 µL was placed on the membrane surface using a syringe. X-ray diffractograms (XRD) of the freeze-dried samples were obtained on an X-ray Powder Diffraction (Malvern™-UK, Empyrean 2). The thermal properties of NC powder and CRC films were measured using thermogravimetric analysis (TGA) (Netzsch TG 209 F3 Tarsus, Germany). The temperature range was 25–1000 °C with a heating rate of 20 °C/min. The optical properties of the CRC films were tested using an Ultraviolet/Visible/Near infrared (UV/Vis/NIR) spectrophotometer (Agilent Technologies Cary 5000-USA series) at a wavelength range of 200 to 1200 nm, equipped with a Diffuse Reflectance (integrating sphere) Accessory DRA-2500 (Labsphere^®^-USA), following the ASTM1003-13 standard method [26]. Haze was calculated using Equation (1):

(1) $$\mathrm{Haze}\;\%=\left(\frac{T4}{T2}-\frac{T3}{T1}\right)*100\%$$

where T_1_ is the incident light transmittance scan, T_2_ is the total light transmitted by the sample, T_3_ is the scattered light by the instrument, and T_4_ is the light scattered by the instrument and the sample.

The surface topography and roughness of the prepared films were characterized by atomic force microscopy (AFM) using Multimode 8 with ScanAsyst and Nanoscope V controller (Bruker-Nano Santa Barbara, Inc., CA, USA). A small piece of each film was mounted on double-sided carbon tape supported on a steel disc. Samples imaging was done using ScanAsyst mode (Nanoscope 9.7) under ambient air conditions with a silicon tip on a nitride lever (SCANASYST-AIR probe with a spring constant of 0.4 N/m). The Rq and Ra roughness values were obtained by analyzing first-order flattened images using Nanoscope Analysis software (version 3.0). All images were acquired on a 40 × 40 μm area with a scanning rate of 0.902 Hz and a resolution of 592 × 592 pixels. Tensile test was carried out to determine the mechanical properties of film samples using Universal Testing System—Instron 5960 with a 50 N load cell at 0.2 mm/min at room temperature, with reference to the ASTM D638-03 [27]. The samples were cut into a rectangular shape with dimensions of 10 mm in width and 30 mm in length. The breathability or water vapor transmission rate (WVTR) of the CRC films were measured using a water permeability cup (purchased from GARDCO, USA), following the desiccant (CaCl_2_) method of ASTM E96 [28]. WVTR is the equivalent of the weight change of the container through the unit area of membrane per day (g/m^2^/day). CRC films were fixed on top of a cup that was filled with a desiccant (CaCl_2_) and the firmly sealed container was weighed to record the initial weight, then placed in an environmental chamber (with a temperature of 20 °C and humidity of 20%) in an upright position, where continuous measurement of the change in container weight was taken at specific times. To calculate the WVTR, we used Equation (2):

(2) WVTR=ΔM/(A∗t)

where ΔM is the change in weight of the container, including sample and desiccant in (g), t is the time (h) of the measurement, and A is the area of the film exposed to moisture (m^2^).

## 3. Results and Discussion

**Figure 2:**
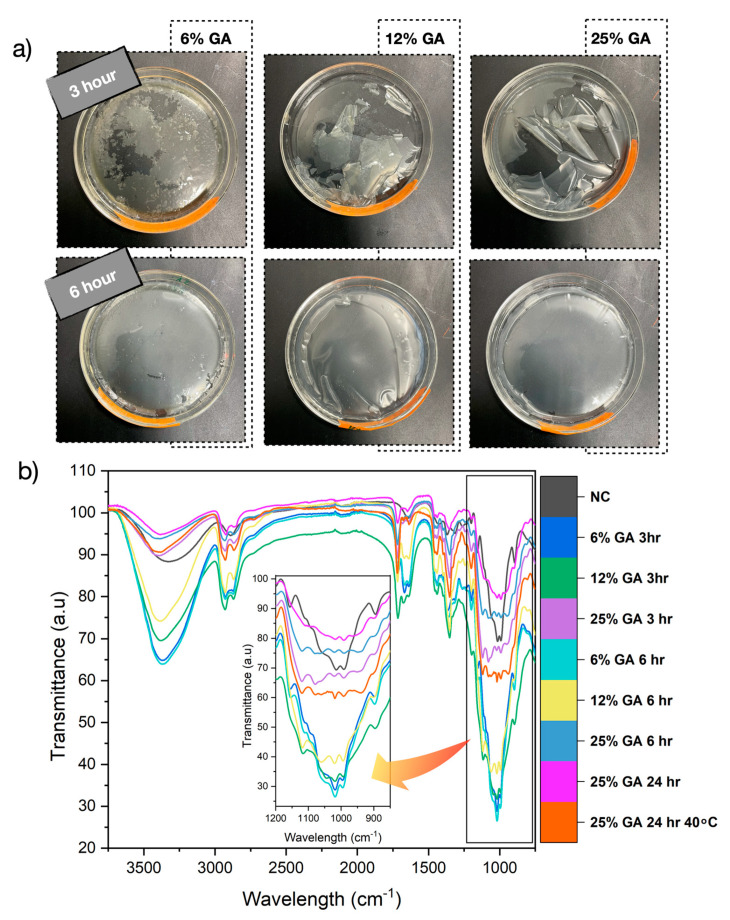
(**a**) Effect of crosslinker concentration and crosslinking time on the formation of crosslinked regenerated cellulose (CRC), (**b**) FTIR spectra of the networked-nanofibrous cellulose (NC) and the CRC films crosslinked with 6, 12, and 25% GA for 3, 6, and 24 hr all at curing temperature of 25 °C (room temperature), in addition to FTIR spectra at curing temperature of 40 °C. The inset represents the magnification of FTIR spectra between 1250–1750 cm^−1^ wavelength.

**Figure 3:**
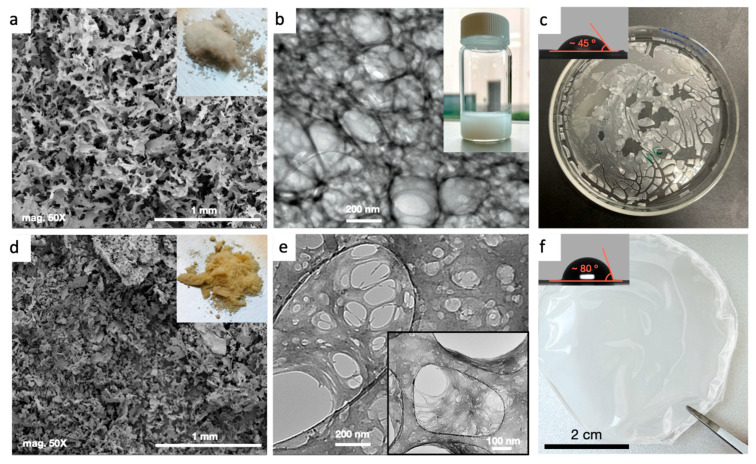
(**a**) SEM image of freeze-dried NC material with inset of a digital image of the powder, (**b**) TEM image of the NC suspension with inset of a digital image of the suspension, (**c**) digital image of the NC film after suspension is cast and dried with inset image of the water contact angle of the dried film, (**d**) SEM image of freeze-dried CRC material crosslinked with 25% GA for 24 hr (CRC 25% 24 hr) with inset of a digital image of the powder, (**e**) TEM image of the CRC-25%-24 hr suspension cured at 25 °C for 1 hr, with the inset of TEM for the CRC-25%-24 hr suspension cured at 40 °C for 1 hr, and (**f**) digital image of the CRC-25%-24 hr film after suspension is cast and dried with inset image of the water contact angle of the dried film.

During hydrolysis, the ability to target and break down the amorphous regions of cellulose at an increased rate compared with the crystalline regions is valid as long as the sulfuric acid concentration is below 64% [29]. Higher concentrations would result in the complete dissolution of cellulose chains due to disruption of the crystalline structure. Thus, it is a narrow window between the dissolution of the crystalline region and the hydrolysis of cellulose to work within. Networked–nanofibrous cellulose was produced via controlled acid-hydrolysis; cellulose chains were dissolved with minimal degradation from the acid by working with a 70% acid solution at a low temperature (5 °C). The crosslinking reaction of the regenerated cellulose suspension was performed to produce films because the mother solution, once cast, suffers from excessive shrinkage upon drying and hence, is not able to form a continuous film. Figure 2a shows the effects of crosslinking time, crosslinking concentration, and curing temperature on film formation. Crosslinking of the regenerated cellulose for 3 hr was not enough to produce a film, even with a high glutaraldehyde concentration (~25%). Increasing the crosslinking time to 6 hr vastly improved film formation; however, some defects were present for all crosslinking concentrations. Crosslinking the regenerated cellulose for an extended time (24 hr) using a 25% glutaraldehyde concentration was the optimum condition to fabricate defect-free film (Figure 3f). FTIR analysis was performed to confirm the chemical reaction with glutaraldehyde. As shown in Figure 2b, the FTIR spectra of NC is compared with the 6, 12, and 25% GA-crosslinked regenerated cellulose (CRC) with different crosslinking reaction times (3, 6, and 24 hr) and curing temperatures of 25 and 40 °C. The peaks of absorption at around 3000–3500, 2750–3000, and 1000–1200 cm^−1^ refer to the OH groups, C–H stretching, and C–O–H and C–O–C asymmetric stretching, respectively. An increase in the intensity of –OH peaks (3000–3500 cm^−1^) for CRC crosslinked with 6 and 12% GA, indicates insufficient crosslinking. A reduced trend in absorption of –OH peaks in 25% GA-crosslinked regenerated cellulose compared with NC was observed by increasing the crosslinking reaction time, suggesting successful crosslinking bonds [30]. The absorption peak at 1115 cm^−1^ is highly evident in the crosslinked film compared with the pristine NC, which is assigned to the ether bond formation between cellulose and GA [31]. At 12 and 25% GA concentrations of the crosslinking agent, the later peak seems to broaden and split, suggesting high degrees of crosslinking. The –OH groups being consumed and the –C–O–C groups increasing in peak broadness are signs of the crosslinking process. The degree of broadening of the –C–O–C peak of the samples cured at 40 °C was intensified compared with the 25 °C-cured samples, suggesting greater crosslinking and densification of the resultant films.

Scanning electron microscopy (SEM) imaging was performed on freeze-dried samples of NC and CRC crosslinked with 25% GA for 24 hr, images are presented in Figure 2. The NC material was found to be a fluffy powder, as seen in Figure 3a. From the TEM images in Figure 3b, it is evident that the NC material possess a network structure with fibers of 30 nm. Networking usually occurs when cellulose chains break and randomly entangle and bundle together. In the regeneration step, the acid is exchanged with the non-solvent (ethanol), causing the chains to bond to neighboring ones aimlessly and precipitate. The combination of acid concentration with a low temperature motivated the networking structure, and this structure was maintained when the regenerating solvent was added at the monitored time. With quick precipitation, the chain bonding was limited to short lengths. The final regenerated cellulose solution is a water suspension solution shown in the inset of Figure 3b. Once the suspension is cast on a glass petri dish and dried, a shrinking and flakiness is noticed (Figure 3c); the formation of a continuous film was not possible. The shrinkage is an indication of the three-dimensional network, as reported before [23]. In Figure 3d, the SEM image of the CRC-25%-24 hr freeze-dried powder shows a denser structure, indicating that the cellulose chains packed densely due to the chemical bonds formed. The same observation was made when the TEM images were examined (Figure 3e). The open structure of NC, although reduced due to the crosslinking reaction, kept its three-dimensional integrity. Figure 2e shows the 25% GA film crosslinked for 24 hr and cured at 25 °C for one hour (before solidification); the inset in the same figure represents the crosslinked film cured at 40 °C for one hour. Evidently, the curing temperature increases the efficiency of crosslinking, leading to closely packed chains. This crosslinking reaction enabled the successful formation of continuous, defect-free film, presented in Figure 3f.

**Figure 4:**
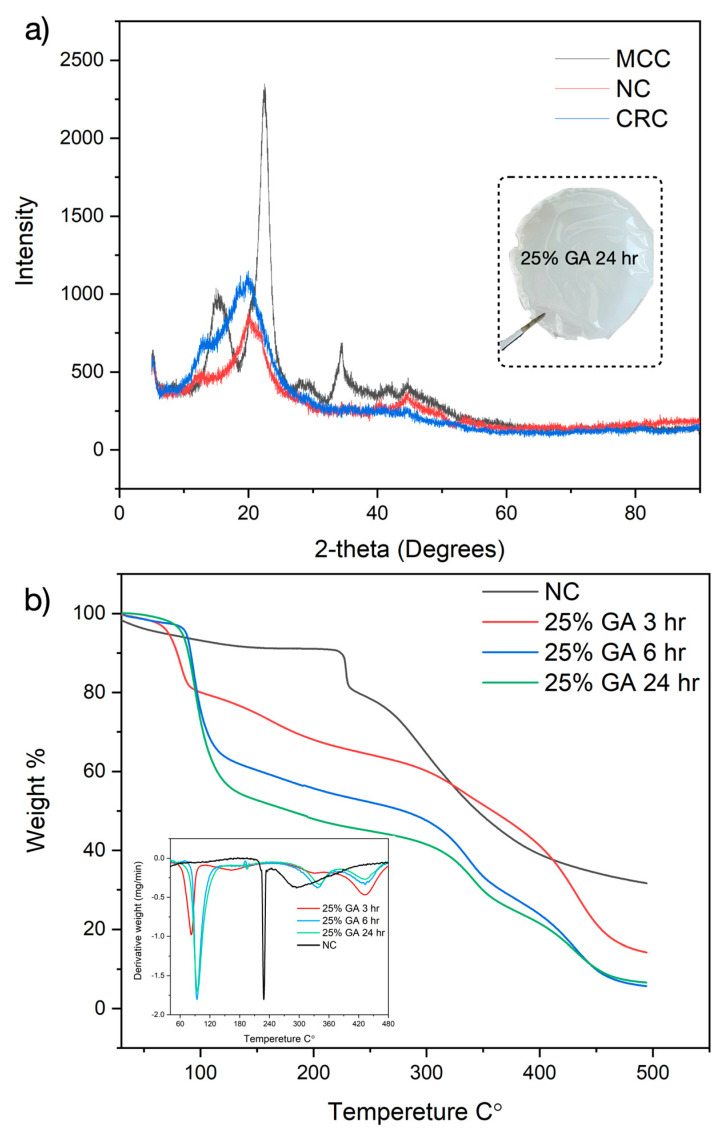
(**a**) XRD analysis of MCC (mother material), freeze-dried NC, and freeze-dried CRC-25%-24 hr with inset digital image of 25% GA-crosslinked NC for 24 hr, (**b**) TGA and DTG curves of NC and CRC crosslinked for 3, 6, and 24 hr using 25% GA with inset representing the DTG curves.

X-ray diffraction data of the NC freeze-dried material alongside the mother material, microcrystalline cellulose (MCC), and the CRC-25%-24 hr are presented in Figure 4a. The data reveals a clear phase shift represented by the different MCC and NC signal patterns. The XRD signal of MCC is a typical crystalline cellulose I signal. On the other hand, the XRD pattern of NC exhibits a less-ordered, amorphous cellulose pattern with characteristics of cellulose II, indicating that the controlled hydrolysis changed the crystallinity of the cellulose. In comparison with NC, the XRD of CRC-25%-24 hr shows that the crosslinked material kept the structure of cellulose II (mainly amorphous nature); however, the characteristic peaks seem to have intensified. Cellulose II-characteristic peaks seem to exist in the CRC samples well, suggesting that crosslinking did not affect the crystallinity or amorphous nature of the cellulose film.

The thermal stability of 25% GA-crosslinked NC and CRC films were studied using thermogravimetric analysis. The TGA and DTG curves of films were compared and are presented in Figure 4b. From the DTG curves, the maximum weight loss sparked at 224 °C for NC material. The low degradation temperature for NC (in comparison with the mother material MCC, which was 348 °C) [23] is a sign that the hydrolysis caused a larger number of free-end chains decomposes at lower temperature [32]. The thermal behavior of the CRC films was different than that of NC and exhibited a higher degradation temperature. It is well-established that chemical crosslinking enhances thermal stability; this observation has been reported elsewhere [33]. The two degradation temperatures for CRC films were 345 and 450 °C. It was also observed that CRC films exhibited a peak below 100 °C, as a result of the evaporation of adsorbed water.

**Figure 5:**
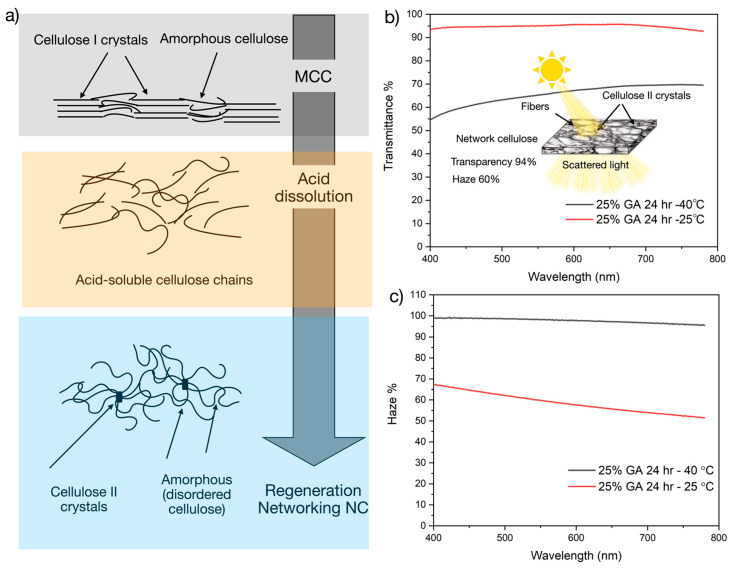
Optical properties of CRC cured at 25 °C and CRC cured at 40 °C. (**a**) Schematic representation of the suggested networking mechanism. (**b**) Transmittance and (**c**) transmission haze of CRC films cured at 25 and 40 °C, with sketch of haze representation in the inset of (**b**). (**d**) Digital images of laser light pointed at and scattered from CRC films cured at 25 and 40 °C. (**e**) Transparent and hazy CRC films cured at 25 and 40 °C, in close contact with and 1 cm above letters written to represent the films’ light transmittance and haze. (**f**) Transmission haze vs. optical transmittance of our CRC films in comparison with other reported cellulose-based transparent and hazy films.

Cellulose, in general, consists of a tangled fiber network which has a blurred appearance with strong light scattering behavior, rendering a transmission haze. Networked–nanofibrous cellulose has a hazy structure, as seen in Figure 3c. The hazy appearance emerged from the disruption that took place during hydrolysis. This disruption introduced changes in the cellulose structure that led to a networking crystallization. The interaction between cellulose and acid can be described as follows (Figure 5a): (i) The acid dissolution starts by opening up the cellulose structure, breaking the hydrogen bonds between chains. The extent of the hydrolysis at given acid concentrations proceeds depending on the reaction time and temperature. (ii) Running hydrolysis at a low temperature of 5 °C slows down the hydrolysis reaction. (iii) Adding anti-solvent at that point withdraws the acid from between cellulose chains and provokes phase separation; the cellulose chains are forced to bundle and re-crystallize in a random manner into the more stable cellulose II with a three-dimensional structure. This is confirmed by XRD data in Figure 4a and TEM image in Figure 3b. As previously reported, the acid concentration, temperature, and mixing time factors all affect the outcome, as such factors target amorphous and crystalline regions at different rates [29,34]. According to previous studies, ethanol was found to be the regenerating solvent with the highest yield [23]. We believe that the network structure of NC is responsible for its hazy properties, as the cellulose II crystals formed during recrystallization can scatter light; the sketch in the inset of Figure 5b displays this scattering mechanism [35]. The microscale crystals in combination with the short fiber bundles formed by the hydrolysis grant the NC its dual optical transparency and hazy properties. Although the controlled hydrolysis treatment initially produces a resilient material, NC suffers from excessive shrinkage once dried, which makes it extremely challenging to prepare films.

Crosslinking of polymers is a well-established method to improve chemical, thermal, and mechanical properties. Glutaraldehyde is a popular crosslinking agent known for its ability to crosslink cellulose under acidic conditions, due to the ability of dialdehydes to enhance resilience, thermal stability, and mechanical properties of cellulose fibers [36]. Glutaraldehyde is considered a successful crosslinking agent because of its multicomponent nature, as a variety of forms can be present in equilibrium at a given pH in the reaction solution [37]. The solubility of GA in water facilitates the crosslinking to take place in aqueous media, which is considered a safe and economic approach [38]. CRC films express high transparency, which is a result of its densely packed homogeneous structure acquired by successful crosslinking. We believe that crosslinking cellulose with GA can combine the haziness from the networked–nanofibrous cellulose with the enhanced transparency from the dense, crosslinked films. A denser fiber network has been shown to perpetuate high optical transmittance [17].

In our study, the CRC films presented a highly transparent cellulose film with a transmission haze of about ~60% at 550 nm (Figure 5b,c) by coupling the high optical transmittance of GA–cellulose dense films and high haze of networked–nanofibrous cellulose through a chemical crosslinking reaction, which retained 94% transparency and 60% transmission haze. The dense structure of the obtained film permits light to go through, whereas the nanosized crystals in the CRC film caused by crystallization provoke strong forward-light scattering. Figure 5b,c also highlight the effect of curing time on the optical properties of the CRC films; curing at 40 °C, for example, increased the hazy nature to almost 100%, but sacrificed the transparency to 65%. This effect may be attributed to the increased degree of crosslinking associated with a high curing temperature, which is confirmed by the FTIR test in Figure 2b.

**Figure 6:**
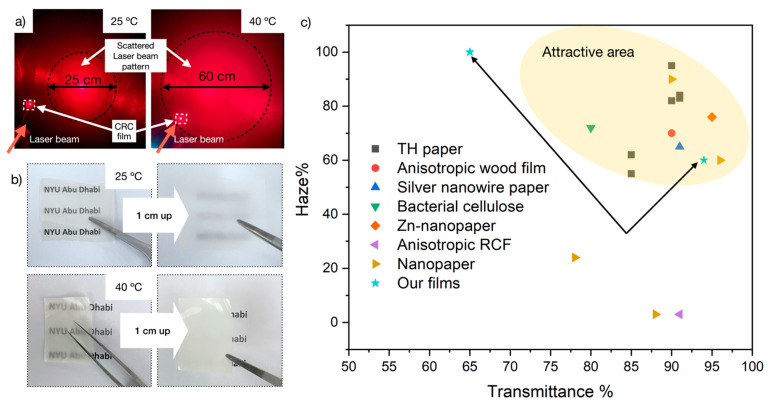
Optical properties of CRC cured at 25 and 40 °C. (**a**) Digital images of laser light pointed at and scattered from CRC films cured at 25 and 40 °C. (**b**) Transparent and hazy CRC films cured at 25 and 40 °C, in close contact with and 1 cm above letters written to represent the films’ light transmittance and haze. (**c**) Transmission haze vs. optical transmittance of our CRC films in comparison with other reported cellulose-based transparent and hazy films.

Figure 6a,b present digital images of the films at 25 and 40 °C with a laser beam being scattered, as well as digital images of the films against NYU Abu Dhabi-written paper (close to and 1 cm above), where the transparent and hazy properties are clearly showcased; the digital images manifest remarkable light scattering. Figure 6c displays the transparency percentage plotted against the transmission haze of the CRC films in comparison with other transparent and hazy cellulose films. Cellulose-based substrates, such as transparent and hazy paper (TH paper), are made from wood fibers or a composite of wood paper and carboxymethyl cellulose [13,15,18,21,39], wood film (anisotropic) [40], Ag-nanowire transparent paper [41], bacterial cellulose film [42], Zn-nanopaper [43], anisotropic regenerated cellulose fiber (RCF) [44], and nanopaper [22,45,46]. Our CRC film transmission haze and transparency data fits within an attractive range for solar cell applications.

**Figure 7:**
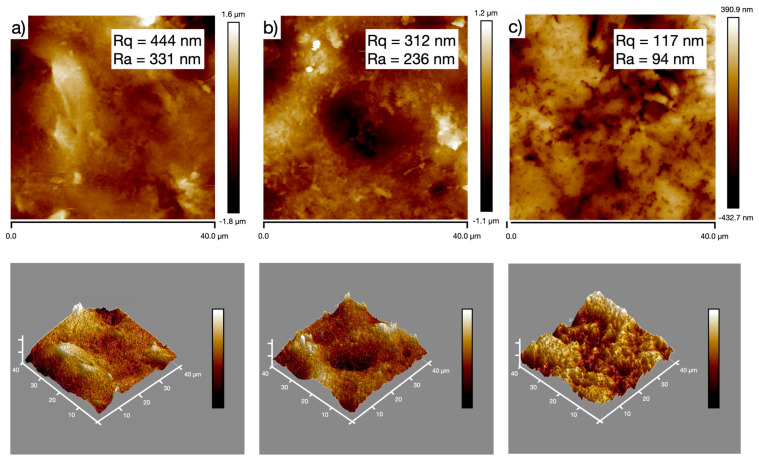
AFM images of the surface of: (**a**) NC, (**b**) CRC cured at 40 °C, and (**c**) CRC cured at 25 °C.

The surface morphology of films influences their optical haze properties. In this study, atomic force microscopy (AFM) was used to characterize the top-view morphology of CRC films (Figure 7). As shown in Figure 7a, the neat NC film has a rough surface, and the root-mean-square roughness (Rq) was recorded at 444 nm. The roughness reported here is an ideal value to create hazy films. In addition, NC is transparent; however, the mechanical stability of NC limits its application. In efforts to improve its mechanical stability, chemical crosslinking was performed. Upon crosslinking, CRC films had better film formation, but showed decreased roughness (Figure 7b,c). This indicates that crosslinking slightly reduces haze properties. Crosslinking connects polymer chains together, which changes the network structure of NC, hence altering its haze properties. Dense CRC films effectively limits light scattering, which endows a higher transparency but reduced haze. The effect of crosslinking temperature was investigated as well; roughness appeared to increase as temperature increased. Rq was recorded at 312 and 117 nm for CRC films crosslinked at 40 and 25 °C, respectively. When a higher temperature is employed, the kinetics of the reaction are induced, meaning there is not enough time for the chains to organize, hence resulting in a rough surface. The crosslinking and drying occurred simultaneously, and evaporation of the solvent (water) was faster at 40 °C, which translated into a rough surface. Haze % was higher at 40 °C (Figure 5c), which is in agreement with roughness values (Figure 7b).

**Figure 8:**
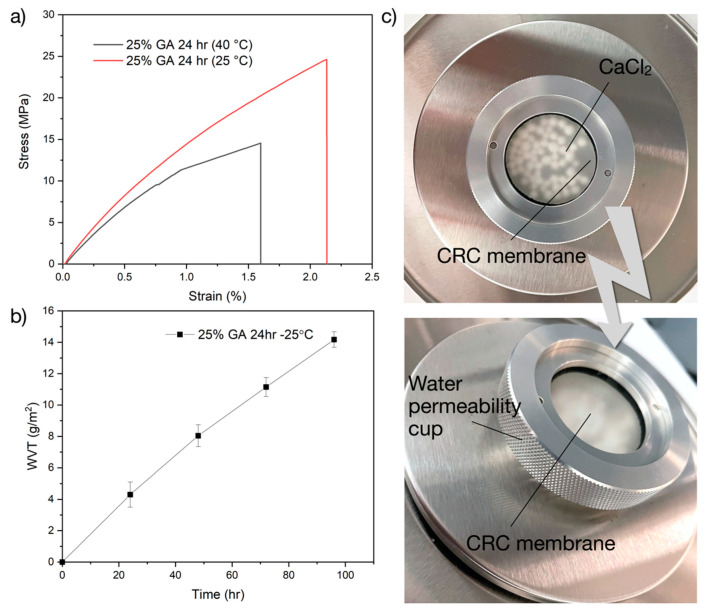
(**a**) Stress-strain curve for the CRC film crosslinked with 25% GA for 24 hr. (**b**) Water vapor transmission rate for 25% GA-CRC films crosslinked for 24 hr (curing at 25 °C). (**c**) Water permeability cup fixed with 25 °C-cured CRC-25% GA for 24 hr as a film, and CaCl_2_ as a desiccant.

The mechanical properties of crosslinked regenerated cellulose films were significantly improved with chemical crosslinking. GA-crosslinked films exhibited good integrated mechanical characteristics; Figure 8a presents the stress–strain curve for the CRC film. Films crosslinked with 25% GA for 24 hr and cured at 25 °C exhibited a tensile strength and elongation at break range of 25 MPa and 2.23%, respectively. This is a significant improvement because the native NC mechanical properties are impossible to obtain due to the shrinkage and flakiness of NC. Similar improvements in the mechanical integrity of crosslinked cellulose nanofibers have been reported in previous studies [30]. In comparison, films crosslinked and cured at 40 °C experienced brittleness and were less mechanically stable (tensile strength of 13 MPa, elongation at break range of 1.6%). Curing of films at higher temperatures will indeed increase the degree of crosslinking; however, it increases the brittleness of the films. Cellulose has strong hydrogen bonding caused by the hydroxyl groups interacting at equatorial positions of a chair-like conformation pyranose ring. A detailed study of such interactions was clearly demonstrated by Bergenstrahle et al. [47]. Past observations have indicated that hydrophobic interactions are influential on the behavior of cellulose in aqueous solutions: this suggest that there is a clear splitting of polar –OH and nonpolar –CH patches in the cellulose structure, thus exhibiting clear amphiphilicity [48]. As a result, the cellulose chains stack via hydrophobic interactions. Crosslinking of the regenerated cellulose imparts increased hydrophobicity to the film structure, as the crosslinking reaction boosts the hydrophobic region causing high resistivity to water vapor (humidity) passage. This is also confirmed by the water contact angle, which increased from 45° in the NC film to 80° in the CRC film, presented in the inset of Figure 3f. Breathability of the CRC films refer to the water vapor transmission rate (WVTR) of GA-crosslinked regenerated cellulose under certain conditions (22 °C, 30% humidity). Higher WVTR values indicates that water vapor passes through the films with less effort. Solar cells require minimal water vapor passage and a high humidity resistance. Herein, the resilient WVTR of CRC was investigated using the upright desiccant (CaCl_2_) method with a water permeability cup (Figure 8c), and the related curves are presented in Figure 8b. The WVTR of the CRC films was calculated to be 3.77 g/m^2^/day, which is considered a good resistivity to humidity for solar cell applications [49].

## 4. Conclusions

A novel cellulose film with high transparency and transmission haze was created using a combination of controlled acid-hydrolysis and chemical crosslinking. The acid-hydrolysis of microcrystalline cellulose (the raw material) was carried out under specified, controlled conditions of acid concentrations and reaction temperatures. Such controlled conditions resulted in a networked, nanofibrous, and opaque cellulose material that would be highly advantageous for optoelectronic applications. Chemical crosslinking was performed to improve the mechanical stability and allow film formation. The prepared films exhibited 94% transparency and 60% transmission haze at 550 nm with a curing temperature of 25 °C. The reason for the high haze arose primarily from the nanoscale crystals and the network nanofibrous nature formed during the controlled recrystallization and regeneration of cellulose, whereas the high transmittance was acquired from the dense packing during chemical crosslinking. Moreover, the roughness of the surface of the films played an important role in light scattering, causing the high optical haze. Increasing the curing temperature to 40 °C resulted in higher optical haze; however, it was at the expense of transparency. In addition, the crosslinked film displayed good mechanical and thermal stability, as well as high water resistivity. Such easy to scale-up, highly transparent, and high haze biodegradable films can be utilized as potential substrates for optoelectronics devices, such as photovoltaic solar cells.

## Data Availability

Not applicable.

## References

[1] Pirani S. (2018). Burning Up: A Global History of Fossil Fuel Consumption.

[2] Shafiee S., Topal E. (2009). When will fossil fuel reserves be diminished?. Energy Policy.

[3] Pizzini S. (2010). Towards solar grade silicon: Challenges and benefits for low cost photovoltaics. Sol. Energy Mater. Sol. Cells.

[4] Sharma S., Jain K.K., Sharma A. (2015). Solar Cells: In Research and Applications—A Review. Mater. Sci. Appl..

[5] Rance W.L., Burst J.M., Reese M.O., Meysing D.M., Wolden C.A., Gessert T.A., Garner S., Cimo P., Barnes T.M. The use of Corning^®^ Willow™ glass for flexible CdTe solar cells. Proceedings of the 2013 IEEE 39th Photovoltaic Specialists Conference (PVSC).

[6] Fobare D., Haldar P., Efstathiadis H., Metacarpa D., Wax J., Olenick J., Olenick K., Venkateswaran V. Novel application of Yttria Stabilized Zirconia as a substrate for thin film CIGS solar cells. Proceedings of the 2014 IEEE 40th Photovoltaic Specialist Conference (PVSC).

[7] Fonrodona M., Escarré J., Villar F., Soler D., Asensi J.M., Bertomeu J., Andreu J. (2005). PEN as substrate for new solar cell technologies. Sol. Energy Mater. Sol. Cells.

[8] Ding J.M., de la Fuente Vornbrock A., Ting C., Subramanian V. (2009). Patternable polymer bulk heterojunction photovoltaic cells on plastic by rotogravure printing. Sol. Energy Mater. Sol. Cells.

[9] Li X., Li P., Wu Z., Luo D., Yu H.-Y., Lu Z.-H. (2021). Review and perspective of materials for flexible solar cells. Mater. Rep. Energy.

[10] Petrov M., Lovchinov K., Mews M., Leendertz C., Dimova-Malinovska D. (2014). Optical and structural properties of electrochemically deposited ZnO nanorod arrays suitable for improvement of the light harvesting in thin film solar cells. J. Phys. Conf. Ser..

[11] Zhai S., Zhao Y., Zhao H. (2019). High-Efficiency Omnidirectional Broadband Light-Management Coating Using the Hierarchical Ordered–Disordered Nanostructures with Ultra-Mechanochemical Resistance. ACS Appl. Mater. Interfaces.

[12] Malinowski C., He F., Zhao Y., Chang I., Hatchett D.W., Zhai S., Zhao H. (2019). Nanopatterned silk fibroin films with high transparency and high haze for optical applications. RSC Adv..

[13] Yang W., Jiao L., Liu W., Dai H. (2019). Manufacture of Highly Transparent and Hazy Cellulose Nanofibril Films via Coating TEMPO-Oxidized Wood Fibers. Nanomaterials.

[14] Lin C., Wang Q., Deng Q., Huang H., Huang F., Huang L., Ni Y., Chen L., Cao S., Ma X. (2019). Preparation of highly hazy transparent cellulose film from dissolving pulp. Cellulose.

[15] Hu W., Chen G., Liu Y., Liu Y., Li B., Fang Z. (2018). Transparent and Hazy All-Cellulose Composite Films with Superior Mechanical Properties. ACS Sustain. Chem. Eng..

[16] Zhu H., Parvinian S., Preston C., Vaaland O., Ruan Z., Hu L. (2013). Transparent nanopaper with tailored optical properties. Nanoscale.

[17] Fang Z., Zhu H., Yuan Y., Ha D., Zhu S., Preston C., Chen Q., Li Y., Han X., Lee S. (2014). Novel Nanostructured Paper with Ultrahigh Transparency and Ultrahigh Haze for Solar Cells. Nano Lett..

[18] Hou G., Liu Y., Zhang D., Li G., Xie H., Fang Z. (2020). Approaching Theoretical Haze of Highly Transparent All-Cellulose Composite Films. ACS Appl. Mater. Interfaces.

[19] Kim J.Y., Lee J.-W., Jung H.S., Shin H., Park N.-G. (2020). High-Efficiency Perovskite Solar Cells. Chem. Rev..

[20] Kim M., Alfano A., Perotto G., Serri M., Dengo N., Mezzetti A., Gross S., Prato M., Salerno M., Rizzo A. (2021). Moisture resistance in perovskite solar cells attributed to a water-splitting layer. Commun. Mater..

[21] Hu W., Fang Z., Liu Y., Zhou Y., Kuang Y., Ning H., Li B., Chen G., Liu Y. (2018). Protonation Process to Enhance the Water Resistance of Transparent and Hazy Paper. ACS Sustain. Chem. Eng..

[22] Chen S., Song Y., Xu F. (2018). Highly Transparent and Hazy Cellulose Nanopaper Simultaneously with a Self-Cleaning Superhydrophobic Surface. ACS Sustain. Chem. Eng..

[23] Hashaikeh R., Abushammala H. (2011). Acid mediated networked cellulose: Preparation and characterization. Carbohydr. Polym..

[24] Samad Y.A., Asghar A., Lalia B.S., Hashaikeh R. (2013). Networked cellulose entrapped and reinforced PEO-based solid polymer electrolyte for moderate temperature applications. J. Appl. Polym. Sci..

[25] Aburabie J., Lalia B., Hashaikeh R. (2021). Proton Conductive, Low Methanol Crossover Cellulose-Based Membranes. Membranes.

[26] Geometries B., Abrasion S. (2012). Standard test method for haze and luminous transmittance of transparent plastics. ASTM Int..

[27] Testing A.S.f. (2010). Materials. Standard Test Method for Tensile Properties of Plastics.

[28] Testing A.S.f. (2013). Materials. Standard Test Methods for Water Vapor Transmission of Materials.

[29] Revol J.-F., Godbout L., Dong X.-M., Gray D.G., Chanzy H., Maret G. (1994). Chiral nematic suspensions of cellulose crystallites; phase separation and magnetic field orientation. Liq. Cryst..

[30] Jeon J.G., Kim H.C., Palem R.R., Kim J., Kang T.J. (2019). Cross-linking of cellulose nanofiber films with glutaraldehyde for improved mechanical properties. Mater. Lett..

[31] Shao L.-L., An Q.-F., Ji Y.-L., Zhao Q., Wang X.-S., Zhu B.-K., Gao C.-J. (2014). Preparation and characterization of sulfated carboxymethyl cellulose nanofiltration membranes with improved water permeability. Desalination.

[32] Staggs J.E.J. (2006). Discrete bond-weighted random scission of linear polymers. Polymer.

[33] Guo B., Chen W., Yan L. (2013). Preparation of Flexible, Highly Transparent, Cross-Linked Cellulose Thin Film with High Mechanical Strength and Low Coefficient of Thermal Expansion. ACS Sustain. Chem. Eng..

[34] Dong X.M., Revol J.-F., Gray D.G. (1998). Effect of microcrystallite preparation conditions on the formation of colloid crystals of cellulose. Cellulose.

[35] Caixeiro S., Peruzzo M., Onelli O.D., Vignolini S., Sapienza R. (2017). Disordered Cellulose-Based Nanostructures for Enhanced Light Scattering. ACS Appl. Mater. Interfaces.

[36] Xu G.G., Yang C.Q., Deng Y. (2004). Combination of bifunctional aldehydes and poly(vinyl alcohol) as the crosslinking systems to improve paper wet strength. J. Appl. Polym. Sci..

[37] Migneault I., Dartiguenave C., Bertrand M.J., Waldron K.C. (2004). Glutaraldehyde: Behavior in aqueous solution, reaction with proteins, and application to enzyme crosslinking. BioTechniques.

[38] Rojas J., Azevedo E. (2011). Functionalization and crosslinking of microcrystalline cellulose in aqueous media: A safe and economic approach. Int. J. Pharm. Sci. Rev. Res..

[39] Zhou P., Zhu P., Chen G., Liu Y., Kuang Y., Liu Y., Fang Z. (2018). A study on the transmission haze and mechanical properties of highly transparent paper with different fiber species. Cellulose.

[40] Zhu M., Wang Y., Zhu S., Xu L., Jia C., Dai J., Song J., Yao Y., Wang Y., Li Y. (2017). Anisotropic, Transparent Films with Aligned Cellulose Nanofibers. Adv. Mater..

[41] Preston C., Fang Z., Murray J., Zhu H., Dai J., Munday J.N., Hu L. (2014). Silver nanowire transparent conducting paper-based electrode with high optical haze. J. Mater. Chem. C.

[42] Wang S., Li T., Chen C., Kong W., Zhu S., Dai J., Diaz A.J., Hitz E., Solares S.D., Li T. (2018). Transparent, Anisotropic Biofilm with Aligned Bacterial Cellulose Nanofibers. Adv. Funct. Mater..

[43] Li X., Zhang X., Yao S., Chang H., Wang Y., Zhang Z. (2020). UV-blocking, transparent and hazy cellulose nanopaper with superior strength based on varied components of poplar mechanical pulp. Cellulose.

[44] Ye D., Lei X., Li T., Cheng Q., Chang C., Hu L., Zhang L. (2019). Ultrahigh Tough, Super Clear, and Highly Anisotropic Nanofiber-Structured Regenerated Cellulose Films. ACS Nano.

[45] Zhu H., Fang Z., Wang Z., Dai J., Yao Y., Shen F., Preston C., Wu W., Peng P., Jang N. (2016). Extreme Light Management in Mesoporous Wood Cellulose Paper for Optoelectronics. ACS Nano.

[46] Yang W., Jiao L., Min D., Liu Z., Dai H. (2017). Effects of preparation approaches on optical properties of self-assembled cellulose nanopapers. RSC Adv..

[47] Parthasarathi R., Bellesia G., Chundawat S.P.S., Dale B.E., Langan P., Gnanakaran S. (2011). Insights into Hydrogen Bonding and Stacking Interactions in Cellulose. J. Phys. Chem. A.

[48] Medronho B., Duarte H., Alves L., Antunes F., Romano A., Lindman B. (2015). Probing cellulose amphiphilicity. Nord. Pulp Pap. Res. J..

[49] Tam H.W., Chen W., Liu F., He Y., Leung T.L., Wang Y., Wong M.K., Djurišić A., Ng A.M.C., He Z. Stability of perovskite solar cells on flexible substrates. Proceedings of the SPIE OPTO.

